# Methylnaltrexone is safe in cancer patients with peritoneal carcinomatosis

**DOI:** 10.1038/s41598-019-44864-2

**Published:** 2019-07-03

**Authors:** Kirbylee K. Nelson, Mark A. Schattner, Robin B. Mendelsohn

**Affiliations:** Department of Medicine, Gastroenterology, Hepatology, and Nutrition Service, Memorial Sloan Kettering Cancer Center, New York, NY USA

**Keywords:** Cancer, Gastroenterology

## Abstract

Opioid-induced constipation (OIC) has become increasingly prevalent with the rise of prescription opioid use and can significantly impact quality of life, especially in patients with advanced illness. Methylnaltrexone has proven effective in treating cancer patients with OIC who have not responded adequately to conventional laxative therapy, though use is relatively contraindicated in those with peritoneal carcinomatosis due to theoretical risk and reported cases of perforation. The aim of this study was to evaluate the safety of methylnaltrexone in patients with carcinomatosis. We performed a retrospective review of 3058 pediatric and adult patients who received methylnaltrexone at Memorial Sloan Kettering Cancer Center from 2009–2016. Data collected included age, cancer diagnosis, history of abdominal surgery, prior radiation therapy, evidence of peritoneal carcinomatosis, and complications. Charts were reviewed for any complications at 24 hours, 72 hours, and one week following drug administration, as well as at present. We identified 3058 patients (median age 56, range 1–95) who received a total of 3995 doses of methylnaltrexone. Three hundred thirty three (median age 55, range 4–88) had peritoneal carcinomatosis. The most common primary malignancies included pancreatic (17.7%), ovarian (13.5%), colon (7.2%), and lung (6.6%). 228/333 (68.4%) had a history of abdominal surgery and 85/333 (25.5%) underwent prior radiation therapy. Three patients had adverse outcomes or complications, with only one (0.3%) thought to be related to methylnaltrexone use. To our knowledge, this is the largest study to evaluate the outcomes of patients with carcinomatosis receiving methylnaltrexone and the first to include pediatric patients. We found one perforation attributed to methylnaltrexone. Methylnaltrexone should be considered for treatment of refractory OIC in cancer patients with peritoneal carcinomatosis due to low risk of complications.

## Introduction

Opioid-induced constipation (OIC) has become increasingly prevalent with the rise of prescription opioid use. Estimates of the prevalence of OIC range from 40% to 95%^1,2^. In contrast to other opioid side effects like nausea and sedation, tolerance does not develop to bowel dysfunction and constipation^3^. Severe OIC may limit opioid therapy, decreasing analgesia, and substantially compromising quality of life, especially in patients with advanced cancer.

Methylnaltrexone bromide (Relistor, Salix Pharmaceuticals), an opioid antagonist approved by the U.S. Food and Drug Administration in 2008, has proven effective in treating cancer patients with OIC who are receiving palliative care and have not responded to conventional laxative therapy. The drug acts as a peripherally-acting mu-opioid receptor antagonist in the gastrointestinal (GI) tract, decreasing the constipating effects of opioids without impacting opioid-mediated analgesic effects on the central nervous system (CNS). The ability to act specifically on peripheral mu receptors is due to the drug’s high polarity, low lipid solubility, and therefore limited potential for CNS penetration^3^.

While the two initial randomized, double-blind, placebo-controlled safety trials for methylnaltrexone only showed mild adverse reactions such as abdominal pain, flatulence, nausea, dizziness, diarrhea, and hyperhidrosis, post-marketing cases of GI perforation were reported^4^. GI perforation was reported in adult patients with OIC and advanced illness with conditions associated with localized or diffuse reduction of structural integrity in the wall of the GI tract. Diseases causing compromised structural integrity included peptic ulcer disease, Ogilvie’s syndrome, diverticular disease, infiltrative GI tract malignancies, and peritoneal metastases.

At Memorial Sloan Kettering Cancer Center, we have been using methylnaltrexone in select patients with carcinomatosis. The aim of this study was to evaluate the safety of methylnaltrexone in patients with carcinomatosis.

## Methods

We performed a single center, retrospective chart review of all pediatric and adult patients who received methylnaltrexone at our institution from January 1, 2009 to December 31, 2016. The study was approved by the Memorial Sloan Kettering Cancer Center Institutional Review Board (IRB) prior to initiation of chart review. Since the study was purely retrospective and was not an experiment on humans nor did it use tissue samples, informed consent was waived by the IRB. All methods were carried out in accordance with relevant guidelines and regulations. The pharmacy database was used to identify patients who received methylnaltrexone during the study period. Some patients received more than one dose.

All methylnaltrexone doses were administered subcutaneously. A standard adult dose was 12 mg, with dose reductions for patients with a creatinine clearance of less than 30 mL/min as recommended by the distributor. Pediatric doses were weight based, ranging from 1.6–12 mg.

Demographic data collected included the patients’ age, gender, and cancer diagnosis. Historical data included information about previous abdominal surgery and prior radiation therapy to the abdomen or pelvis. Safety data included evidence of peritoneal carcinomatosis and complications. Charts were reviewed for any complications at 24 hours, 72 hours, and one week following drug administration, as well as at present.

### Definitions

Constipation was defined as any documentation of constipation in the medical record, with no required minimum number of days since the last bowel movement.

Laxative use included therapy with agents such as osmotic laxatives, stool softeners, or stimulants used for any duration of time prior to methylnaltrexone administration.

Peritoneal carcinomatosis was defined as malignancy disseminating and growing in the peritoneal cavity. Carcinomatosis was confirmed by reviewing radiology and pathology reports.

Bowel obstruction was identified radiographically on computed tomography imaging performed prior to methylnaltrexone administration.

### Statistical analysis

We used descriptive statistics to describe the data obtained.

## Results

We identified 3058 patients (median age 56, range 1–95) who received a total of 3995 doses of methylnaltrexone. Three hundred thirty three (median age 55, range 4–88) had peritoneal carcinomatosis. Of those patients with peritoneal carcinomatosis, 208/333 (62%) were women.

The most common primary malignancies included pancreatic (17.7%), ovarian (13.5%), colon (7.2%), and lung (6.6%). Some patients had more than one cancer diagnosis. 228/333 (68.5%) had a history of abdominal surgery and 85/333 (25.5%) underwent prior radiation therapy to the abdomen or pelvis (Table 1).

**Table 1:** Demographic and historical data for patients with peritoneal carcinomatosis.

Number of patients with carcinomatosis	333
**Median age (years)**	55 (range 4–88)
**Gender (male/female)**	125/208
Cancer*	
Head and neck	1
Lung	22
Breast	19
Esophageal	7
Stomach	20
Pancreatic	59
Biliary	7
Appendiceal	7
Neuroendocrine	5
Renal	14
Bladder/Urothelial	11
Ovarian	45
Fallopian tube	3
Endometrial/Uterine	18
Cervical	7
Small bowel	2
Colon	24
Rectal	9
Anal	3
Prostate	3
Sarcoma	16
Central nervous system	20
Melanoma	8
Leukemia	2
Lymphoma	5
Multiple myeloma	2
Unknown primary	10
Abdominal surgery	228
Radiation therapy (to abdomen or pelvis)	85

*some patients had more than one cancer diagnosis.

**Figure 1:**
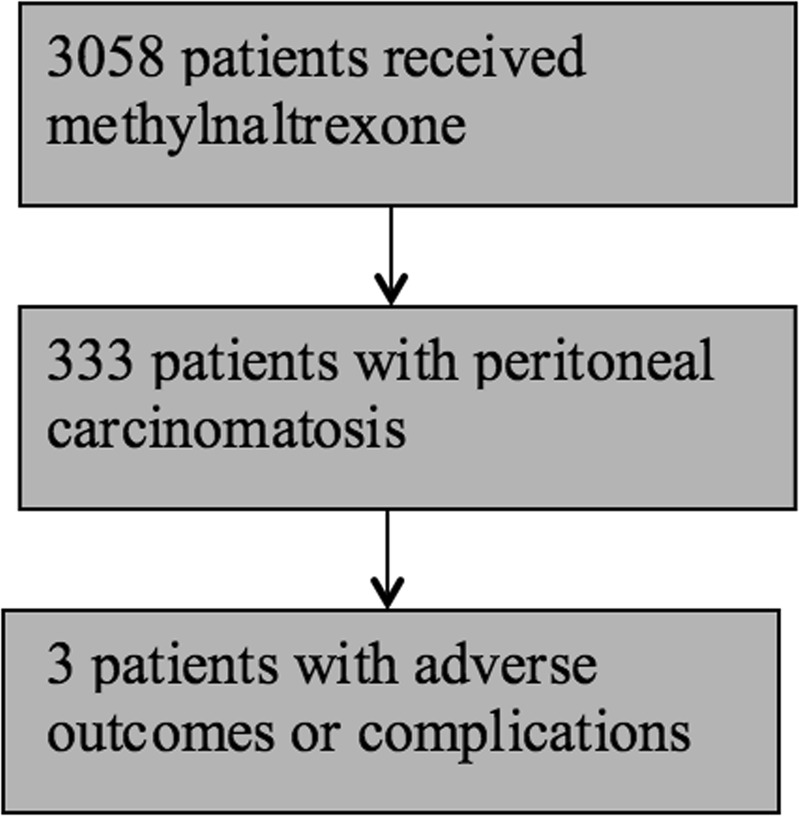
Flow diagram of patients who received methylnaltrexone.

Three patients (0.9%) with peritoneal carcinomatosis had adverse outcomes or complications after methylnaltrexone administration (Fig. 1). One patient was found to have free air nine days after administration, but this was in the setting of worsening distention, clostridium difficile infection, and cecostomy. Another patient developed elevated lactic acid of unclear etiology the day after administration. Neither of the above cases were thought to be due to methylnaltrexone. One patient (0.3%) had a perforation that resulted in death and was attributed to methylnaltrexone. Methylnaltrexone was administered for clinical and radiographic evidence of prominent stool burden throughout the colon despite laxatives, with resultant worsening abdominal pain and distention. Computed tomography showed a new frank bowel perforation with large volume pneumoperitoneum, fecal material in the peritoneal cavity, and diffusely thickened small bowel with enhancing peritoneal reflections consistent with peritonitis. The patient was treated with broad-spectrum antibiotics, however given poor overall prognosis, was made comfort care and died within 36 hours (Table 2).

**Table 2:** Adverse outcomes or complications associated with methylnaltrexone use.

	Age	Gender	Cancer	Abdominal surgery	Radiation therapy	Adverse outcomes or complications	Timing after dose
1	58	F	pancreatic	yes	no	Free air (in the setting of worsening distention, clostridium difficile, and cecostomy)	9 days
2	84	F	pancreatic	no	no	Elevated lactic acid	1 day
3	57	F	pancreatic	yes	no	Perforation	1 day

## Discussion

OIC is a frequently occurring and distressing side effect in patients with progressive cancer at the end of life. The debilitating adverse effects of OIC can be severe enough to seriously impact patients’ quality of life comparable even to pain, to where some prefer inadequate pain control to avoid these symptoms. Even when laxatives are taken concurrently with opioids, approximately half of patients treated for OIC do not achieve the desired improvement^1,2^.

Although methylnaltrexone has proven to effectively treat OIC in both adult and pediatric patients, it is relatively contraindicated in cases of peritoneal carcinomatosis due to reported cases of perforation with intestinal obstruction^5,6^. While the exact mechanism of GI perforation associated with methylnaltrexone use is not clear, it is possibly due to a prokinetic effect that goes beyond reversing opioid-induced hypomotility combined with compromised integrity of the GI tract. There were seven post-marketing cases of GI perforation involving methylnaltrexone in the Adverse Event Reporting System (AERS) database in patients with anatomical or pathological abnormalities in the upper or lower GI tract^7^.

Despite post-marketing cases of perforation, a systemic review and meta-analysis by *Siemens et al*. showed methylnaltrexone to be effective and safe with only rare occurrence of serious adverse events, none of which included perforation^8^. Review of the seven studies included only showed an incidence of methylnaltrexone-related serious adverse events of 0.2% (4/1860). These included extra-systoles, cardiovascular collapse related to dehydration from diarrhea, flushing, and delirium. The most frequent adverse events included abdominal pain, nausea, and diarrhea.

Our study included 3058 patients who received a total of 3995 doses of methylnaltrexone. Of the 3058 total patients, 333 had peritoneal carcinomatosis. The most common primary malignancies, in order from most to least common, included pancreatic, ovarian, colon, and lung. Of the 333 patients, 68.5% had a history of abdominal surgery and 25.5% underwent prior radiation therapy to the abdomen or pelvis. Three patients had adverse outcomes or complications after methylnaltrexone administration, however only one, a perforation, was related to drug administration. The other two cases were not suspected to be related.

To our knowledge, this is the largest study to evaluate the outcomes of patients with carcinomatosis receiving methylnaltrexone and the first to include pediatric patients. We found one perforation attributed to methylnaltrexone in this large cohort, therefore inferring methylnaltrexone is a safe therapy in the acute setting. Methylnaltrexone should be considered for treatment of refractory OIC in cancer patients with peritoneal carcinomatosis due to low risk of complications.
